# Ex vivo MRI cell tracking of autologous mesenchymal stromal cells in an ovine osteochondral defect model

**DOI:** 10.1186/s13287-018-1123-7

**Published:** 2019-01-11

**Authors:** Hareklea Markides, Karin J. Newell, Heike Rudorf, Lia Blokpoel Ferreras, James E. Dixon, Robert H. Morris, Martin Graves, Joshua Kaggie, Frances Henson, Alicia J. El Haj

**Affiliations:** 1 Institute of Science and Technology in Medicine, Guy Hilton Research Centre, Keele University, Thornburrow Drive, Stoke-on-Trent, ST4 7QB UK; 2 Department of Chemical Engineering, Healthcare Technologies Institute, Birmingham University, B15 2TT, Birmingham, UK; 3 Department of Surgery, University of Cambridge, Addenbrooke’s Hospital, Hills Road Cambridge, Cambridge, CB2 0QQ UK; 4 Department of Veterinary Medicine, University of Cambridge, Madingley Rd, Cambridge, CB3 0ES UK; 5 Centre for Biomolecular Sciences, The University of Nottingham, University Park, Nottingham, NG7 2RD UK; 6 School of Science and Technology, Nottingham Trent University, Clifton, Nottingham, NG11 8NF UK; 7 Department of Radiology, University of Cambridge, Hills Rd, Cambridge, CB2 0QQ UK

**Keywords:** SPIONs, MRI, Tracking, Osteochondral, Mesenchymal stromal cells, Pre-clinical. Translational

## Abstract

**Background:**

Osteochondral injuries represent a significant clinical problem requiring novel cell-based therapies to restore function of the damaged joint with the use of mesenchymal stromal cells (MSCs) leading research efforts. Pre-clinical studies are fundamental in translating such therapies; however, technologies to minimally invasively assess in vivo cell fate are currently limited. We investigate the potential of a MRI- (magnetic resonance imaging) and superparamagnetic iron oxide nanoparticle (SPION)-based technique to monitor cellular bio-distribution in an ovine osteochondral model of acute and chronic injuries.

**Methods:**

MSCs were isolated, expanded and labelled with Nanomag, a 250-nm SPION, and using a novel cell-penetrating technique, glycosaminoglycan-binding enhanced transduction (GET). MRI visibility thresholds, cellular toxicity and differentiation potential post-labelling were assessed in vitro. A single osteochondral defect was created in the medial femoral condyle in the left knee joint of each sheep with the contralateral joint serving as the control. Cells, either GET-Nanomag labelled or unlabelled, were delivered 1 week or 4.5 weeks later. Sheep were sacrificed 7 days post implantation and immediately MR imaged using a 0.2-T MRI scanner and validated on a 3-T MRI scanner prior to histological evaluation.

**Results:**

MRI data demonstrated a significant increase in MRI contrast as a result of GET-Nanomag labelling whilst cell viability, proliferation and differentiation capabilities were not affected. MRI results revealed evidence of implanted cells within the synovial joint of the injured leg of the chronic model only with no signs of cell localisation to the defect site in either model. This was validated histologically determining the location of implanted cells in the synovium. Evidence of engulfment of Nanomag-labelled cells by leukocytes is observed in the injured legs of the chronic model only. Finally, serum c-reactive protein (CRP) levels were measured by ELISA with no obvious increase in CRP levels observed as a result of P21-8R:Nanomag delivery.

**Conclusion:**

This study has the potential to be a powerful translational tool with great implications in the clinical translation of stem cell-based therapies. Further, we have demonstrated the ability to obtain information linked to key biological events occurring post implantation, essential in designing therapies and selecting pre-clinical models.

## Background

The treatment of osteochondral lesions (OCLs) remains a burdensome clinical problem significantly impacting the life of the patient with substantial costs to the health-care system [1]. OCLs present as injuries to the cartilage surface of an articular joint, penetrating the subchondral bone [2]. A number of possible aetiologies have been identified with repetitive micro-trauma as the leading cause, affecting people of all ages [3–5]. The likeness of such injuries self-repairing is limited due to the inherently poor healing capacity of hyaline cartilage despite evidence of short-term repair responses [6]. If left untreated, these injuries can progress in severity and lead to further degeneration of the articular surface, ultimately resulting in osteoarthritis (OA). Once an injury has reached this stage, symptoms and treatment options become increasingly severe and challenging. Emphasis is therefore placed on early intervention to prevent progression of focal lesions to advanced cartilage degeneration and OA [7, 8].

Novel cell-based therapies are currently under development and aim to address this clinical need with the use of mesenchymal stromal cells (MSCs) leading research efforts [4]. MSCs are multipotent stem cells residing within specialised 3D microenvironments of connective tissues which are able to differentiate towards tissues of the mesenchymal lineage (cartilage, bone and fat). The premise of osteochondral tissue engineering involves the use of an osteochondral mimicking scaffold embedded with MSCs which can be implanted directly to the site of injury to initiate repair [9]. This typically involves highly invasive and lengthy surgeries to prepare the injured site for implantation and, as of yet, has failed to generate adequate clinical outcomes to support clinical adoption. Alternatively, an injectable cell therapy model whereby MSCs are minimally invasively delivered to the site of injury could create an appealing treatment model [6]. In exploring this mode of delivery and optimising towards clinic adoption, it becomes necessary to gather information on the short-term in vivo events occurring post implantation in terms of accuracy of cell transplantation, bio-distribution and cell integration alongside tissue regeneration [10, 11]. In this way, parameters linked to the risks and successes of such therapies can be evaluated during pre-clinical studies.

Magnetic resonance imaging (MRI)-based cell tracking techniques have been used across a number of tissue engineering strategies to monitor exogenous cell populations in vivo [12]. Target cells are labelled with superparamagnetic iron oxide nanoparticles (SPIONs) either with or without the use of a transfection agent prior to implantation to generate negative or hypointense contrast when MR imaged using T_2_ or T_2_* sequences [13]. This technique has been used to minimally invasively monitor the delivery, retention and engraftment of implanted cell seeded scaffolds in small animal models of cartilage injury and arthritis [1, 10, 14]. The application of SPIONs in regenerative medicine is not limited to their use as contrast agents but also extends to applications of cell activation [15] and site-specific targeting [16]. To achieve this breadth of applications, SPION properties and labelling parameters can be tailored and optimised to suit each technique [17]. Our group has pioneered a bio-magnetic approach, magnetic ion channel activation (MICA), using the commercially available SPION, Nanomag, to activate and drive MSC differentiation towards bone and cartilage lineages [18, 19].

In this study, we aim to investigate the use of Nanomag as a potential MRI contrast agent which can, in later applications, be used as a dual MRI and activation agent in orthopaedic therapies. Furthermore, we define a protocol to successfully label ovine MSCs with Nanomag using a novel cell-penetrating peptide and a technique known as glycosaminoglycan-binding enhanced transduction (GET) to enhance Nanomag uptake [20]. Under these conditions, we demonstrate the feasibility of short-term tracking of labelled cells by veterinary MRI scanner whereby cells are minimally invasively delivered. We further demonstrate how we can use this short-term method to investigate the behaviour of stem cells following MSC therapy for acute and chronic OA models.

## Methods

Reagents were purchased from Sigma Aldrich unless otherwise specified.

### Ethics

All in vivo experiments were approved by the UK Home Office and Local Ethics committee. Methods were conducted in accordance to the UK Home Office Regulations and protocols approved by University of Cambridge Animal Welfare and Ethical Review Body.

#### Animals

Six mature female Welsh Mountain Sheep were used in this study (*n* = 3 for each model; acute and chronic).

#### Bone marrow harvest

Autologous MSCs were isolated by bone marrow aspiration from the iliac crest of anaesthetised animals using a 100-mm 8 Gauge Jamshidi needle (UK Medical Ltd., Sheffield, UK). The aspirate was collected in αMEM containing 10% FBS, 1% l-glutamine (LG), 1% antibiotic and anti-mycotic (AA) and a heparin sodium solution to prevent clotting (5000 IU/ml, Wockhardt, Wrexham, UK). The aspirate was then transported on ice for downstream MSC isolation.

#### Surgical procedure

The stifle joints of each animal were opened via a parapatellar approach with the animals under general anaesthesia. An 8-mm-diameter, 8-mm-deep, osteochondral defect was created in the medial femoral condyle (MFC) in the left stifle joints of each animal under strict asepsis. The defects were centralised in the medial femoral condyle, aligned with the medial crest of the trochlear groove and 10 mm distal to the condyle groove junction. After surgery, the joints were closed in routine fashion, and the animals were allowed to fully bear weight post-operatively.

#### Cell delivery

**Fig. 1:**
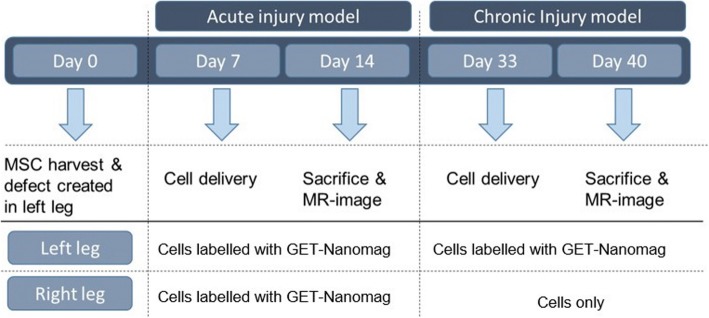
Schematic representation of experimental design

Prior to delivery, GET-Nanomag-labelled cells were stained with CM-DiI (Molecular Probes, Paisley, UK), a fluorescent cell tracker, as per manufacturers’ instructions. 10^7^ labelled cells were subsequently re-suspended in 2 ml serum-free media (SFM) containing 1% LG and1% AA and transported in darkness on ice for subsequent intra-articular delivery. Cells ± Nanomag were injected using a 21-g needle into the left and right femoro-patella joints at different time points as shown in Fig. 1.

#### Sacrifice

Sheep were sacrificed 7 days post cell implantation using an overdose of intravenous anaesthetic solution. Legs were retrieved immediately and frozen for further analysis (MRI and histology).

#### Serum collection

Serum was collected from the jugular vein on day 0 and day 7.

### Cell isolation and expansion

Autologous ovine MSCs were isolated by red blood cell (RBC) lysis treatment. The aspirate was filtered using a 100-μm cell sieve and washed through with SFM prior to centrifuging at 220 g for 30 min. The supernatant was carefully removed, replaced with 5 ml of ice-cold RBC lysis buffer and incubated for a further 3 min at room temperature with gentle agitation. Lysis buffer was quenched with 40 ml of ice-cold PBS and lysed cells removed by centrifugation (220 g; 5 min). This process was repeated until a white pellet appeared at which point 3 ml of media (αMEM media, 20% FBS, 1% l-glutamine and 1% AA) was added and cells transferred to a T25 flask and maintained at 37 °C for 1 week before further media changes. MSCs were subsequently cultured (37 °C and 5% CO_2_) in αMEM expansion media (EM; 10% FBS, 1% l-glutamine and 1% AA) with a single media change in the first week and two media changes per week thereafter until cell had reached passage 2 for all animal experiments.

### Cell labelling

MSCs were labelled with Nanomag-D (Micromod, Germany), a commercially available 250-nm SPION with COOH functionality using the cell-penetrating peptide P21-8R and GET technology (obtained from the University of Nottingham). Cells were labelled at a ratio of 25 μg of Nanomag (1 mg/ml) per 2 × 10^5^ cells and complexed with 1 μl (1 mM) P21-8R per 50 μg Nanomag [20]. In brief, MSCs (P2) were seeded in T175 flasks at 80% confluency in EM and allowed to attach overnight. Media was then replaced with the labelling solution (consisting of EM and the appropriate amount of GET-Nanomag) and cells incubated overnight at 37 °C and 5% CO_2_ to enable efficient internalisation of Nanomag. Following this, cells were washed thoroughly in PBS (3×) to remove non-internalised Nanomag.

### Assessment of Nanomag uptake by Prussian blue staining

Prussian blue is an iron-based stain routinely used to identify the presence of SPIONs. Here, it was implemented to firstly evaluate the efficiency of the cell-penetrating peptide in mediating Nanomag uptake and then to compare uptake across six ovine MSC donors. MSCs were methanol fixed post Nanomag labelling (15 min; RT) then treated with a 1:1 solution of 20% aqueous HCL (hydrochloric acid) and 10% aqueous potassium ferrocyanide (20 min; RT) (*n* = 3). Cells were imaged by light microscopy (EVOS XL Core Cell Imaging System) with bright blue staining revealing the presence SPIONs.

### Particle characterisation

The effect of GET complexing on the hydrodynamic diameter and zeta potential (charge) of Nanomag was assessed using the Malvern Zetasizer Nano ZS. For both charge and size, 10 μl of Nanomag and GET-Nanomag were re-suspended in water and three consecutive measurements (12–15 subruns per repeat) per sample at room temperature were taken.

### Assessment of cell viability and proliferation post Nanomag labelling

Live/dead staining was used to evaluate the extent of cell death post Nanomag and GET-Nanomag labelling (25 μg/ml Nanomag). Labelled MSCs were cultured for either 24 hrs or 5 days then treated with 1% calcein AM and 2% propidium iodide prepared in PBS according to the manufacturer’s instructions for 45 min at 37 °C, whilst protected from light. Samples were imaged using a UV fluorescent microscope (Nikon Eclipse Ti-S). For a quantitative assessment of cell health, alamar blue, a metabolic assay, was carried out according to the manufacturer’s instructions. Here, cells labelled with 1, 20, 25 and 50 μg/ml of either Nanomag or GET-Nanomag were assessed at day 0 (pre-labelling), day 1 and again at day 7 post-labelling for metabolic activity and compared to untreated controls.

### Cell characterisation

Ovine MSCs (P3) from each sheep donor (6 in total) were characterised by their tri-lineage differentiation potential post Nanomag and GET-Nanomag labelling. In all cases, cells were plated in triplicate (10^4^ cells/cm^2^ for osteogenesis and chondrogenesis and 2.5 × 10^4^ cells/cm^2^ for adipogenesis) and allowed to attach overnight. Cells were then labelled with 25 μg/ml Nanomag as described above and treated with the appropriate differentiation induction media.

#### Osteogenesis

Osteogenic induction media consisted of low glucose DMEM (1 g/L), 10% FBS, 1% l-glutamine, 1% AA, 10^− 8^ mM dexamethasone, 0.8 mM l-ascorbic acid and 10 mM β-glycerophosphate*.* Cells were cultured for 21 days with weekly media changes and fixed in 10% neutral buffered formalin (10 min; RT) for subsequent Alizarin red staining (1%).

#### Adipogenesis

Cells were cultured in adipogenic induction media consisting of high-glucose DMEM (4.5 g/L), 1% BSA, 100 μM indomethacin, 1 μm dexamethasone, 0.5 mM IBMX (3-Isobutyl-1-methylxanthine) and 10 μg/ml insulin for 72 hrs. Cells, thereafter, were cultured in adipogenic maintenance media consisting of DMEM (4.5 g/L), 1% BSA and 10 μg/ml insulin for a further 14 days. Cells were fixed in formalin (10 min: RT), and adipogenesis was evaluated by Oil Red O staining.

#### Chondrogenesis

Chondrogenic media consisted of high-glucose DMEM (4.5 g/L), 1% FBS, 1% l-glutamine, 1% AA, 0.1 μm dexamethasone, 50 μg/ml l-ascorbic acid, 10 ng/ml TGF-β1 (Peprotech, UK) and 50 mg/ml ITS (insulin, transferrin, sodium selenite). Media was completely changed every 3 days for 21 days. Chondrogenesis was evaluated histologically by Alcian blue staining. In all cases, control cells were cultured in proliferation media for the duration of the protocol.

### MRI

#### In vitro MRI

The in vitro MRI detection threshold was determined as previously described by Markides et al [10]*.* In brief, Nanomag and GET-Nanomag-labelled cells were encapsulated within a 2 mg/ml rat tail type I collagen hydrogel (BD Biosciences, Oxford, UK) and samples MR imaged using a Brucker 2.3-T animal scanner (Nottingham Trent University) with a multi-slice multi-spin echo (MSME) imaging sequence: TR = 5 s, TE =10.173 ms, matrix size = 256 × 128, spatial resolution = 0.35 × 0.35 mm.

#### Ex vivo MRI 0.25 T

Joints were imaged with a 0.25-T MRI (Esaote). The following sequences were used: T_1_ echo train = 1, TR = 0.0 ms, TE = 26.0 ms, slice thickness = 2.5 mm, dimension size = 2.5 × 2.5 mm^2^, matrix size = 256 × 256, T_2_ echo train = 8, TR = 0.0 ms, TE = 120.0 ms, slice thickness = 4.0 mm, dimension size = 4.4 × 4.4 mm^2^, matrix size = 512 × 512, 3D T_2_-weighted hybrid contrast-enhanced (Hyce) echo train = 1, TR = 0.0 ms, TE = 21.1 ms, slice thickness = 2.5 × 2.5 mm^2^, dimension size = 2.5 × 2.5 mm^2^, matrix size 512 × 512.

#### Ex vivo MRI 3 T

Joints were imaged with a 3D multi-echo spoiled GRE on a 3.0-T MRI (MR750, GE Healthcare), with matrix size = 512 × 332 × 76, with six echo times (TEs = 7.0, 12.7, 18.4, 24.1, 29.7, 35.4 ms), dimension size = 0.37 × 0.37 × 1.5 mm^3^, field of view = 190 × 123 × 114 mm^3^, flip angle = 20°, coil acceleration (asset) = 2.0, and an asymmetric readout = 0.7.

### Quantification of CRP (c-reactive protein) levels

CRP levels were determined 7 days post cell implantation and compared to pre-implantation levels to assess immune response associated with GET-Nanomag delivery. Blood was collected from the jugular vein and decanted into untreated 20-ml falcon tubes (no anticoagulant) immediately prior to cell delivery (day 0) and upon sacrifice (day 7). Serum was collected by allowing blood to coagulate overnight at 4 °C then centrifuged at 2000 *g* for 30 min. CRP levels were determined by ELISA (Neo Bio Labs, USA) according to the manufacturer’s instructions.

### Histology

The distal femoral condyle of each animal, the medial and lateral meniscus and synovial membrane from the cranial and dorsal aspect of the joint were collected post-mortem, decalcified using EDTA and paraffin embedded. Seven-micrometre sections were obtained. Sections were then stained for hematoxylene and eosin (H&E) to identify tissue structure and Prussian blue to determine the presence of Nanomag-labelled cells prior to imaging.

### Statistical analysis

GraphPad Prism V6.0 was used for all statistical analysis. Data is presented as the average value ± standard deviation (S.D.) with statistical significance determined by t-test or two-way ANOVA as appropriate. In all cases, * is *p* < 0.05, ** is *p* < 0.01, *** is *p* < 0.001, **** is *p* < 0.0001 and ns is no significance.

## Results

### GET peptide complexation promotes enhanced uptake of Nanomag by oMSCs across multiple sheep donors

**Fig. 2:**
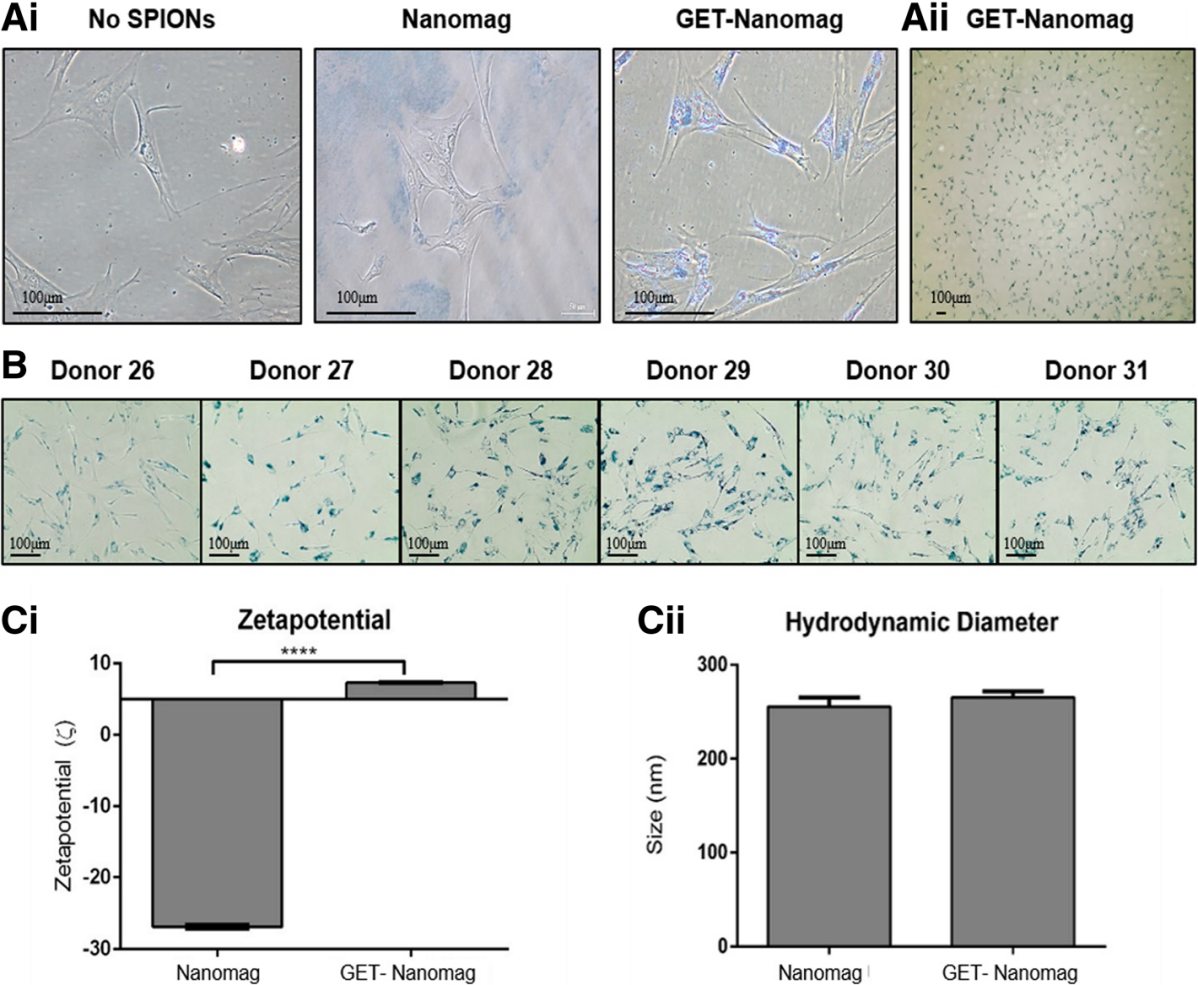
Assessment of Nanomag uptake, cell morphology and Nanomag properties as a consequence of GET complexing. Prussian blue staining highlights the presence of iron-based magnetic nanoparticles by blue staining. **Ai** Prussian blue staining of oMSCs incubated with no SPIONs, Nanomag only and GET-Nanomag. **Aii** Prussian blue staining of GET-Nanomag-labelled oMSCs demonstrating similar uptake by all cells within a single culture well. **B** Prussian blue staining of GET-Nanomag-labelled MSCs from six sheep donors demonstrate consistent uptake across multiple donors. **Ci** Zetapotential measurements of Nanomag and GET-Nanomag determined in water. **Cii** Hydrodynamic diameter of Nanomag and GET-Nanomag determined in water. Data in **Ci** and **Cii** represents the mean zeta potential (ζ) ± s.d (*n* = 3) and hydrodynamic diameter ± s.d (*n* = 3) respectively with significance determined by unpaired *t*-test where **** is *p* < 0.0001. Scale bars = 100 μm

Prussian blue staining for iron content was successful in demonstrating enhanced uptake of Nanomag as a result of GET complexing, complementing previous work [20]. This is clearly shown as intense regional blue staining within internal cell compartments as opposed to naked Nanomag which was located in the extracellular regions of each cell (Fig. 2Ai). Furthermore, cell morphology remained unchanged post GET-Nanomag uptake with similar uptake levels observed within a single culture well (Fig. 2Aii) and across multiple sheep donors (Fig. 2B). Complexing Nanomag with GET further resulted in a significant change in the charge of the particle from − 26.86 ± 0.3 to + 7.29 ± 0.1 (*p* < 0.0001) (Fig. 2Ci) with no significant influence on the hydrodynamic diameter of the particle (Fig. 2Cii).

### No adverse effects on cell viability, proliferation and tri-lineage differentiation potential of oMSCs as a result of GET-Nanomag labelling

**Fig. 3:**
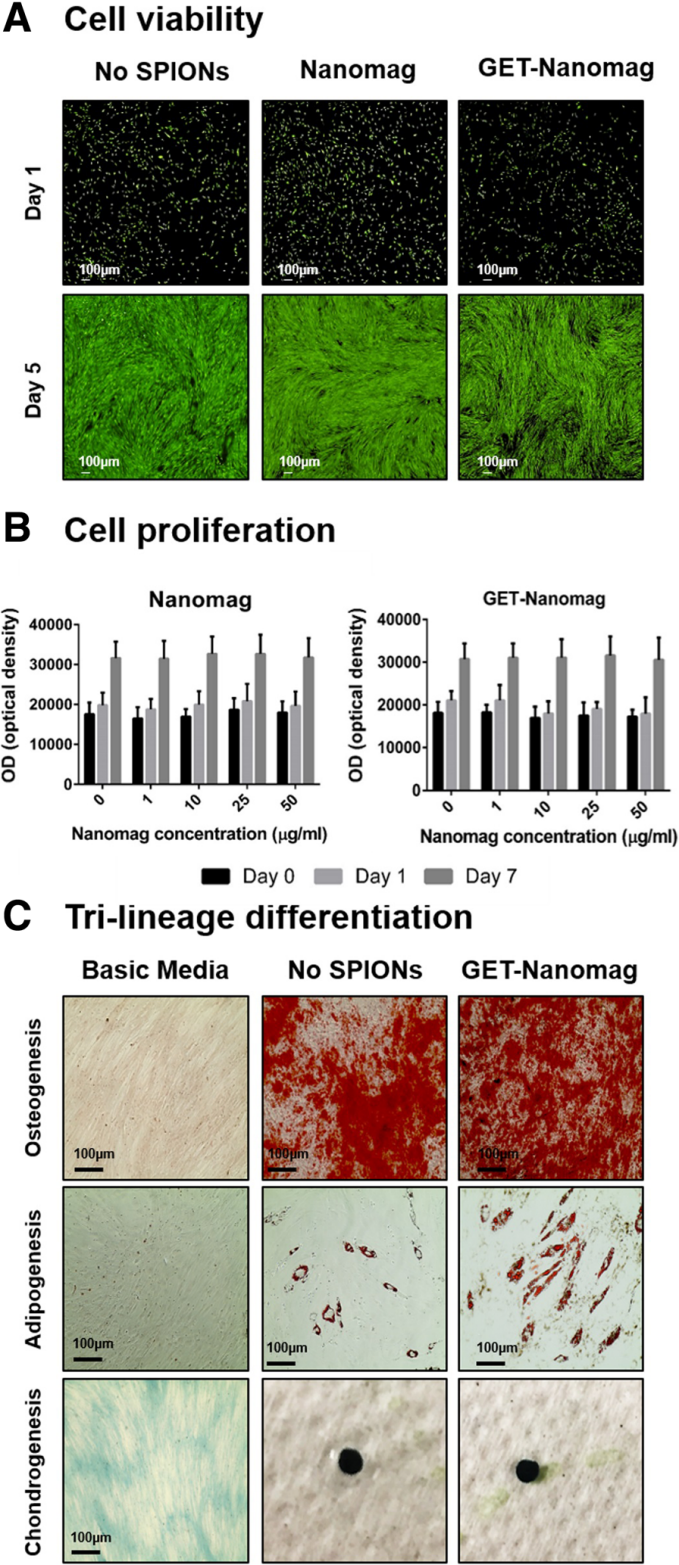
In vitro assessment of cell viability, proliferation and tri-lineage differentiation potential post GET-Nanomag labelling of oMSCs. Images are selected for a single sheep donor but are representative of all donors. **a** Live/dead staining of labelled (25 μg/ml) and unlabelled oMSCs 24 hrs and 5 days post-labelling. **b** Quantification of cellular health via Alamar blue metabolic assay at days 0 (pre-labelling), 1 and 7 (post-labelling) with cells labelled with 0, 1, 10, 25 and 50 μg/ml Nanomag or GET-Nanomag. **c** Tri-lineage differentiation of labelled (25 μg/ml) and unlabelled oMSCs from a representative sheep donor (donor 26) where alizarin red staining was used to confirm osteogenesis (day 28), Oil Red O staining to assess adipogenesis (day 14) and finally Alcian blue staining to evaluate chondrogenesis (day 21) (*n* = 3). GET-Nanomag-labelled cells cultured in basic media served as representative control groups (*n* = 3). Scale bars = 100 μm

Labelling cells either with or without the addition of the cell-penetrating peptide had no adverse impact on the viability of oMSCs in short- (24 hr) and long-term (5 days) cultures as determined by live/dead staining. An obvious increase in the number and density of cells was further observed in all cases over a 5-day culture period implying that labelled cells maintained their ability to proliferate with results equivalent to unlabelled controls (Fig. 3A). Quantitative Alamar blue results further support this data by demonstrating no diminished viability and proliferation potential (as inferred by metabolic activity) for cells labelled either with Nanomag or GET-Nanomag (0, 1, 10, 25 and 50 μg/ml) over 7 days and compared to unlabelled controls. GET-Nanomag-labelled oMSCs were further shown to successfully differentiate towards the osteogenic, adipogenic and chondrogenic lineages when cultured in the relevant differentiation media in a comparable manner to unlabelled cells (Fig. 3C).

### Enhanced MRI contrast observed in vitro as a result of GET-mediated cell labelling

**Fig. 4:**
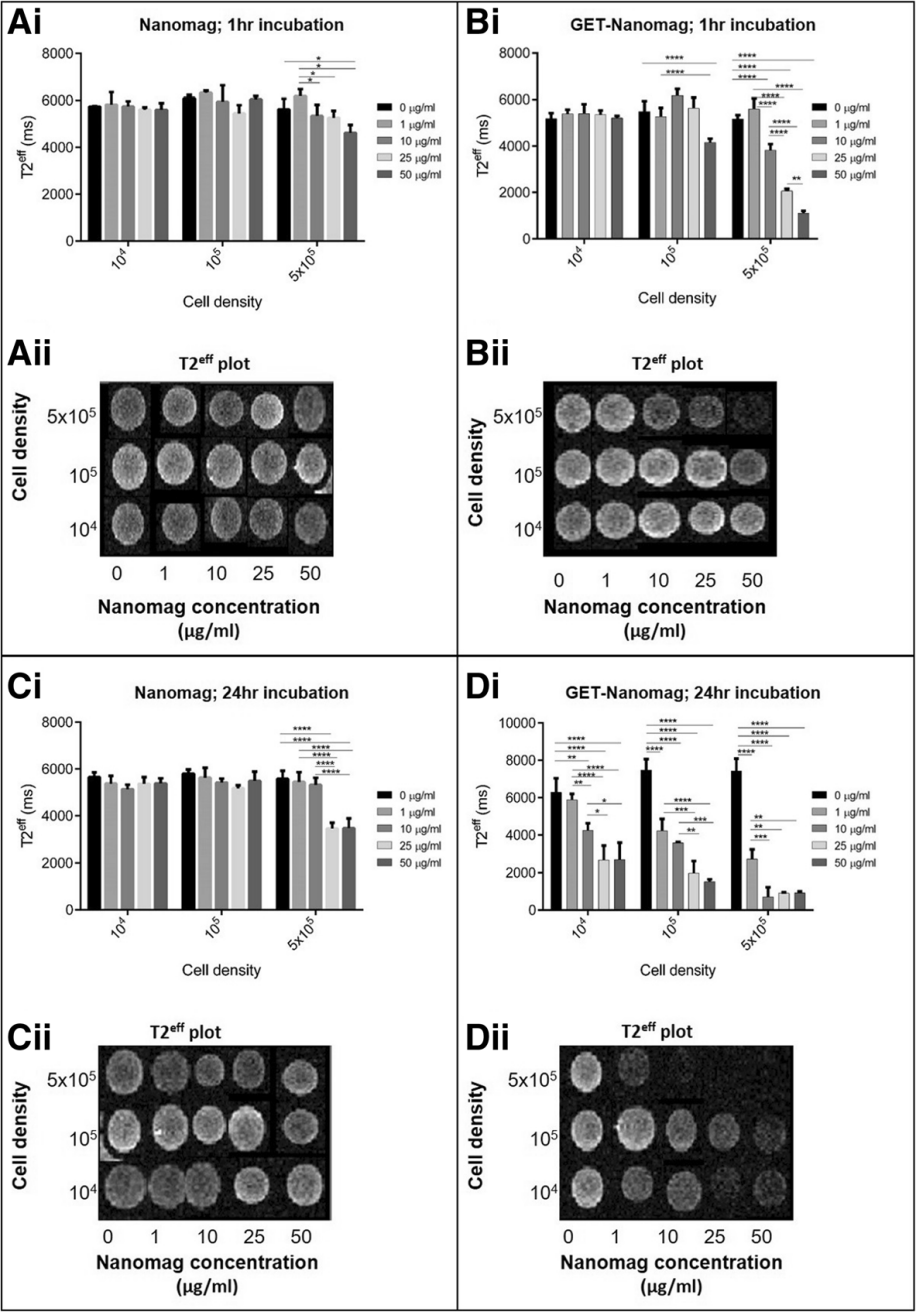
In vitro MRI dose response of Nanomag-labelled MSCs encapsulated in 2.5 mg/ml collagen type 1 gel. **A** and **B** are MSCs labelled with Nanomag for 1 h and 24 hrs respectively. **C** and **D** are MSCs labelled with GET-Nanomag for 1 hr and 24 hrs respectively. For each subfigure, **i** is the T_2_^eff^ measurement produced from the MRI image and **ii** is the corresponding T_2_^eff^ plots. Data represents mean T_2_^eff^ values *±* s.d. (*n* = 3) with significance determined by two-way ANOVA statistical test where * is *p* < 0.05, ** is *p* < 0.01, *** is *p* < 0.001 and **** is *p* < 0.0001

The in vitro MRI visibility threshold in terms of cell dose and Nanomag concentration was assessed in a 3D collagen gel system. Internalised iron-based particles disrupt the local magnetic field causing a shortening of T_2_*. Consequently, this creates hypointense regions of signal void (black areas) on an MRI scan. To quantify this, measurements of T_2_^eff^ are undertaken. T_2_^eff^ is a parameter which is based on T_2_* but more easily measured in the case of short T_2_* as it relies on the generation of a number of consecutive spin echoes. In this study, T_2_^eff^ remained long in groups lacking the GET peptide. Furthermore, increasing incubation time (1 to 24 h), increasing cell dose (10^4^–5 × 10^5^ cells per 100 μl collagen gel) and increasing Nanomag concentration (0–50 μg/ml) had no significant impact on T_2_^eff^ with similar values measured in the control unlabelled cell groups (Fig. 4Ai, Ci). This is further observed visually in the T_2_^eff^ plots where the colour intensity from the grey-scale MRI scans remained unchanged in comparison to the control unlabelled groups (light grey) (Fig. 4Aii, Cii). In contrast, a distinct and significant shortening of T_2_^eff^ is measured with increasing incubation time, cell dose and Nanomag concentration in GET-Nanomag-labelled cell groups (Fig. 4Bi, Di). This is likely due to the improved uptake efficiency of Nanomag as a result of the GET cell-penetrating peptide. Based on the corresponding T_2_^eff^ plots, good contrast was generated (black region) when labelling 5 × 10^5^ cells with 50 μg/ml GET-Nanomag over a 1 h incubation period (Fig. 4Bii). These conditions are improved by increasing the incubation to 24 h resulting in contrast for as low as 10^4^ cells labelled with 25 μg/ml GET-Nanomag or for 5 × 10^5^ cells labelled with 10 μg/ml GET-Nanomag (Fig. 4Dii).

### Surgical model

**Fig. 5:**
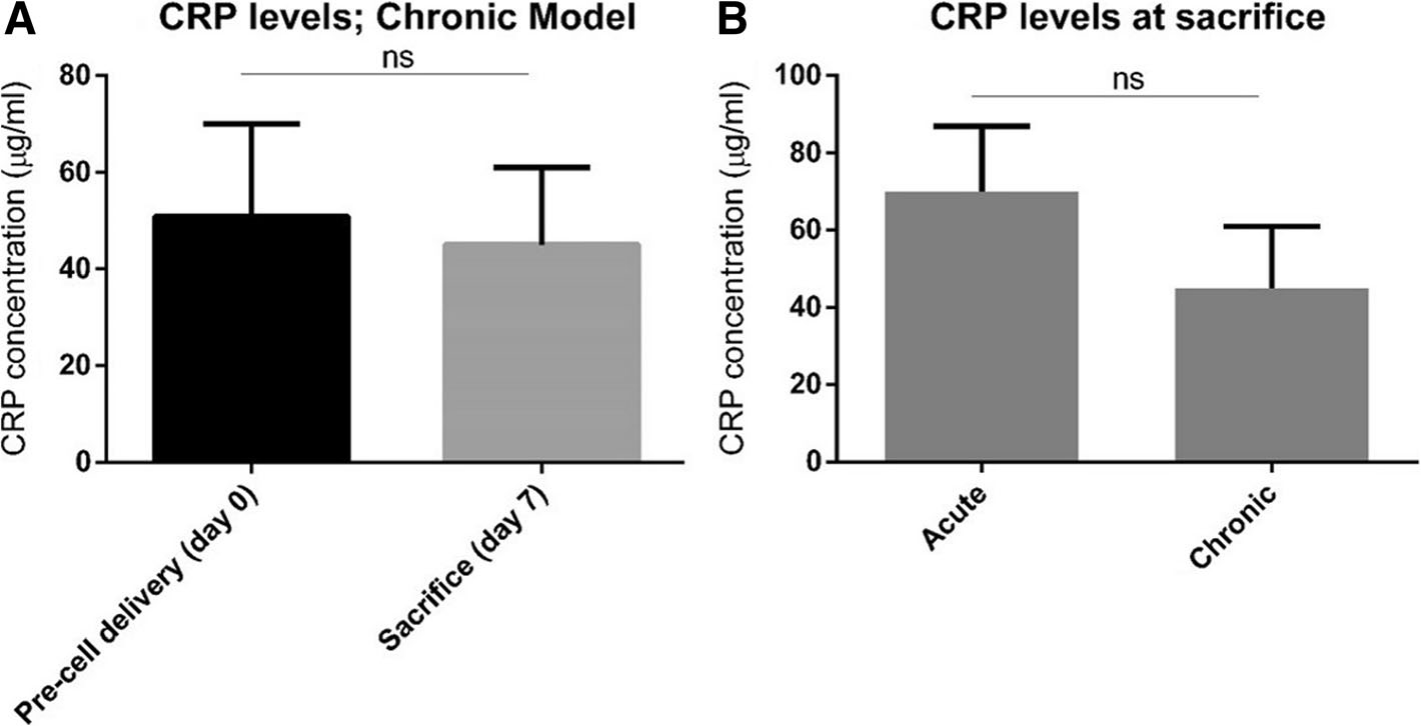
Autologous serum CRP levels. **a** CRP levels in the chronic injury model where levels were measured prior to the delivery and upon sacrifice in three sheep. **b** Comparative CRP levels at sacrifice in the acute and chronic models. Data represents mean CRP levels **±** s.d. for three individual sheep with significance determined by unpaired *t*-test where ns implies no significance

Surgery was tolerated well by all sheep without complications. No signs of an adverse immune reaction to GET-Nanomag delivery in either model were detected. C-reactive protein (CRP) levels were measured on day 0 (pre-cell implantation) and upon sacrifice on day 7 in the chronic model revealing no deviation from baseline levels (Fig. 5a). Furthermore, comparing CRP levels at sacrifice in the acute and chronic models revealed no significant differences. (Fig. 5b).

### Ex vivo MRI tracking

**Fig. 6:**
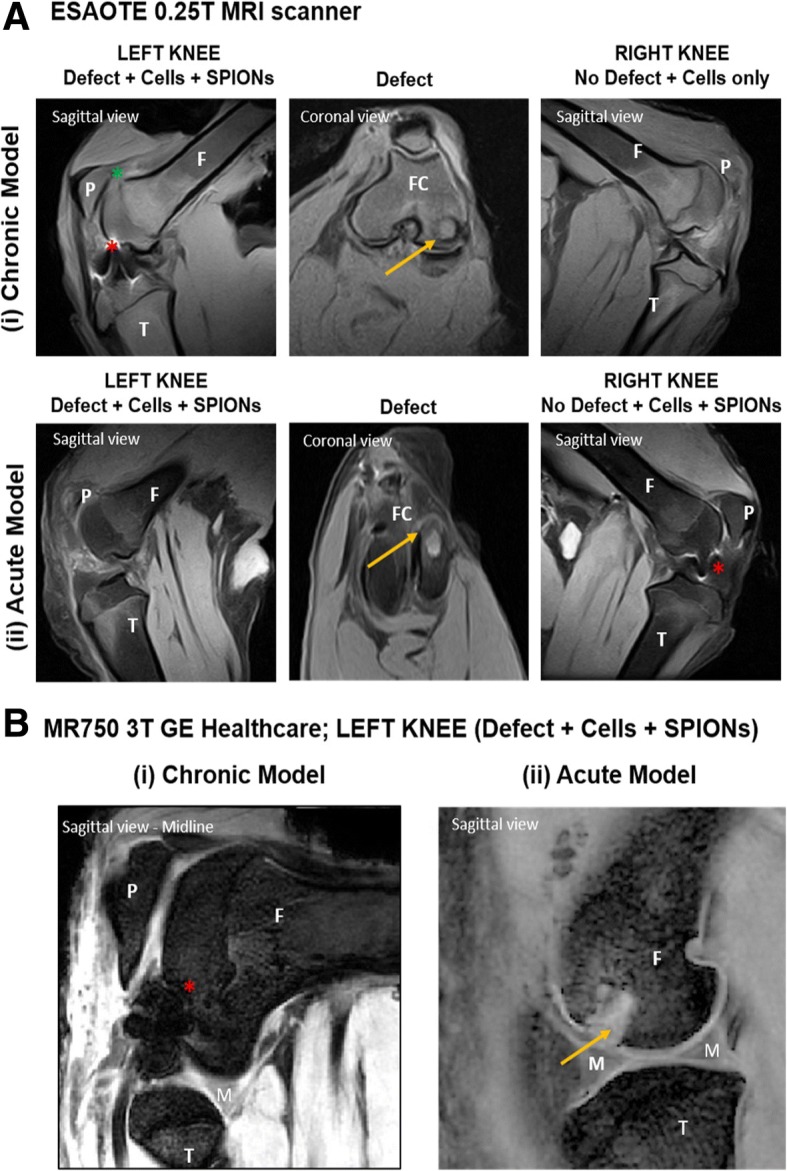
Cross-sectional MRI images of the knee joints 7 days post cell delivery. **a** T_1_ MRI scans obtained using a 0.25-T Esaote MRI scanner and validated using a **b** MR750 3-T GE Healthcare scanner with T_2_ sequences. Red star represents areas of blooming artefact due to the presence of significant amounts of SPION-labelled cells whilst yellow arrow represents the site of the osteochondral defect and green star the site of the femoral patella joint. F = femur, T = tibia, M = meniscus, P = patella, FC = femoral condyle

Knee joints were MR imaged post sacrifice initially on a 0.25-T veterinary MRI scanner (Fig. 6a) and then validated on a 3-T clinical MRI scanner (Fig. 6b). Iron-based magnetic nanoparticles are visualised as hypointense regions of signal void or “black” areas on MRI scans within the knee joint. In this study, the presence of GET-Nanomag-labelled cells is clearly visible as “black” regions (red star) in the injured leg (left knee) of the chronic model (Fig. 6a (i)) and the control leg (right knee) of the acute model (Fig. 6a (ii)). On the contrary, no “black” regions were observed in the injured leg (left knee) of the acute model (Fig. 6a (ii)) nor in the control leg (right knee) (Fig. 6a (i)). Finally, labelled cells were not observed within the osteochondral defect (yellow arrow) in either model via MRI. Similar results are observed in the left legs of the 3-T images (Fig. 6b).

### Histological evaluation

**Fig. 7:**
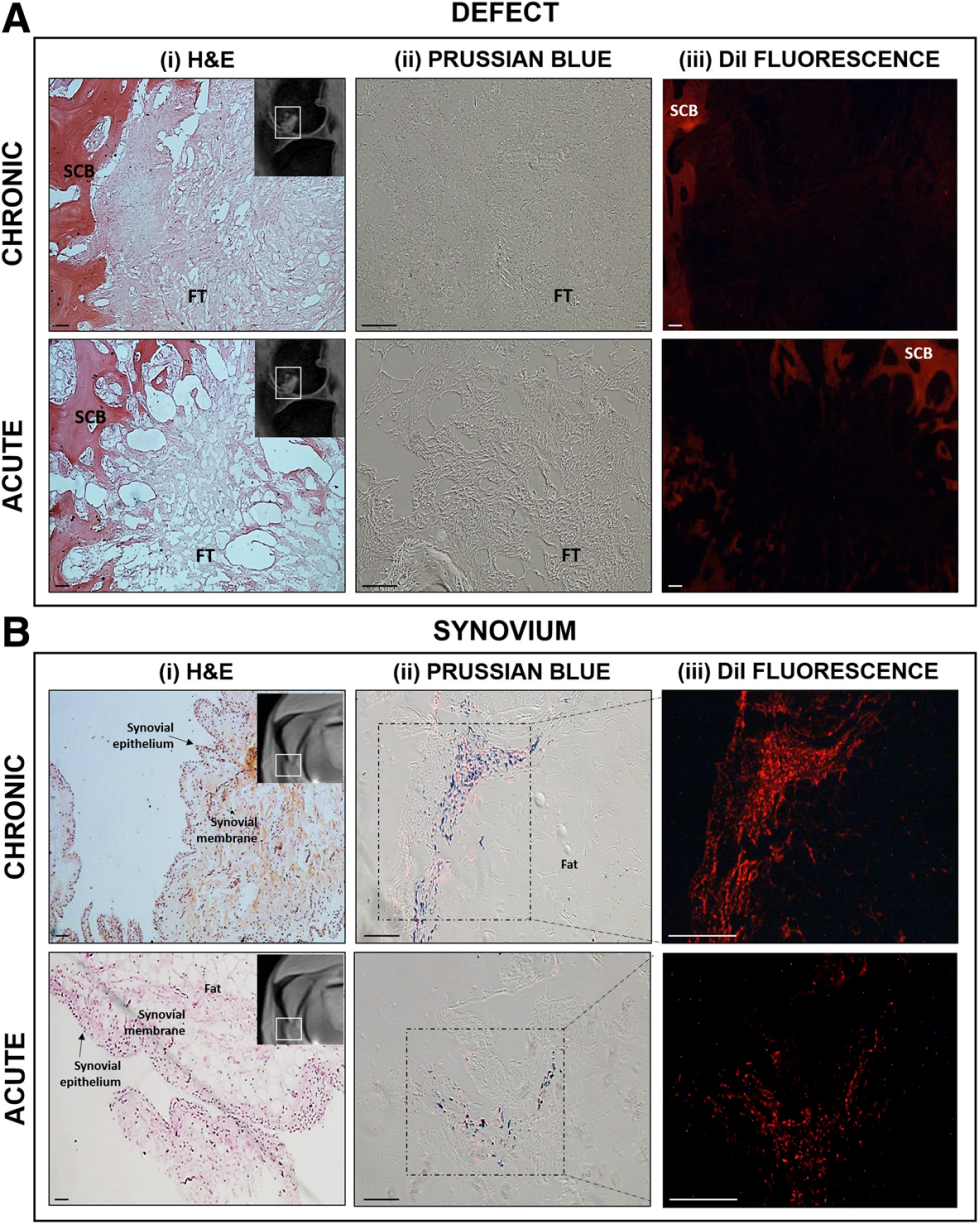
Representative tissue sections showing **a** the osteochondral defect and **b** the synovium from the injured leg (left leg) of both models. Tissue structure is shown by (i) H&E staining where connective tissue is depicted by pink whilst cellular matter is stained purple, insert; MR image depicting general location of histological section (defect and synovium). (ii) Prussian blue staining revealed the presence of iron oxide-based magnetic nanoparticle and is seen as blue staining. (iii) Fluorescent red staining represents delivered oMSCs stained with the membrane dye DiI prior to delivery. Scale bars = 100 μm. SCB = subchondral bone, FT = fibrous tissue

To validate and confirm the location of implanted cells, histological sections of the osteochondral defect and the synovium were scrutinised for the presence of GET-Nanomag-labelled cells. Implanted cells were identified by red fluorescence significant of the DiI stain used to label cells pre-delivery whilst Prussian blue staining was used to identify the iron-based magnetic nanoparticle, Nanomag. H&E staining further revealed key tissue structures and allowed for the defect and synovium to be accurately identified. Fibrous tissue is seen to completely fill each defect of both the chronic and acute model. The matrix appeared to be denser and more organised in the chronic model (Fig. 7a (i)).

No evidence of implanted cells was detected in the osteochondral defect in either model (Fig. 7a (ii), (iii)), consistent with MRI results. Instead, labelled cells are observed within the synovial lining of both the chronic and acute injury models, evident by the overlaying Prussian blue stain and florescent DiI stain (Fig. 7b (ii), (iii)). Comparatively, increased cell density is observed in this region in the injured leg of the chronic model relative to the acute model (Fig. 7b (iii)) with subsequent localisation of SPIONs (Fig. 7b (ii)) implying that cells have retained the SPION label.

**Fig. 8:**
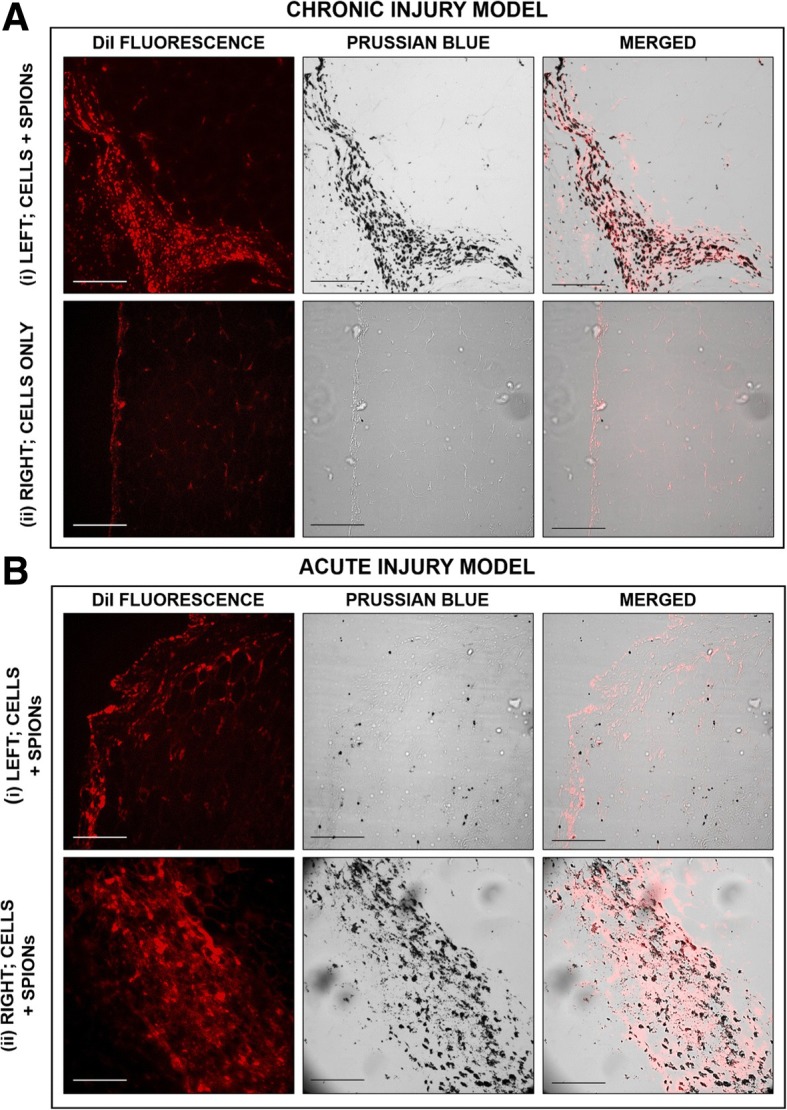
Histological sections of the synovium in the **a** chronic and **b** acute injury models with focus on (i) the left injured leg and (ii) the right non-injured control leg where delivered oMSCs are stained with the membrane dye DiI and are shown by red fluorescent imaging whilst Prussian blue staining identifies Nanomag and is seen as the black staining in these monochrome images. Scale bars = 100 μm

On closer inspection of the synovial lining of the chronic model, significant localisation of labelled cells is observed in injured leg (left leg) and to a lesser extent in the non-injured control leg (right leg) (Fig. 8a). In the acute model, however, areas of higher cell density are observed in the control leg as opposed to the injured leg (left leg) (Fig. 8b).

### Evidence of co-localisation of CD45-positive leukocytes and GET-Nanomag is observed in the synovial lining of injured legs in chronic model only

**Fig. 9:**
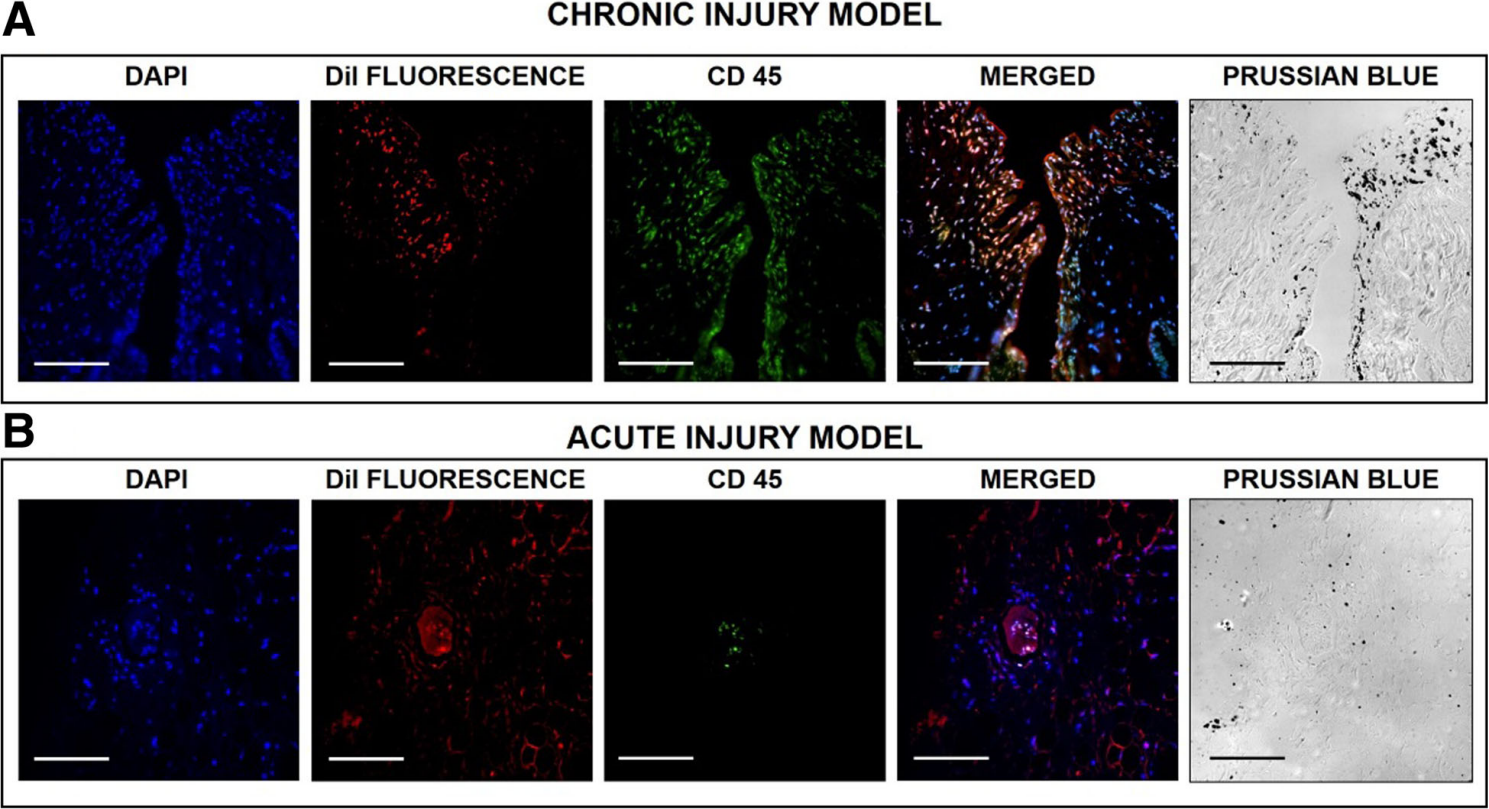
Immunohistochemical analysis at the synovial site 7 days post implantation in the **a** chronic and **b** acute injury models. Blue staining (DAPI) represents cell nuclei whilst red fluorescence is significant of the DiI tag of implanted GET-Nanomag-labelled MSCs. Leukocytes, positive for the CD45 marker, are stained green with Prussian blue staining highlighting the magnetic nanoparticle, Nanomag, and is seen as the black staining in these monochrome images. Scale bars = 100 μm

The presence of leukocytes (white blood cells) was assessed by immunohistochemical staining for CD45. Leukocytes (stained green) are present in both injury models with an obvious increase observed in the chronic model compared to the acute injury model (Fig. 9). Furthermore, a strong correlation in the localisation of DiI-labelled MSCs and leukocytes in the chronic injury model is observed, suggesting that GET-Nanomag-labelled MSCs are potentially engulfed and transported by the leukocytes to the synovium (Fig. 9a).

## Discussion

Despite extensive clinical efforts, cartilage and osteochondral injuries continue to burden the patient and healthcare system. In light of this, cell-based therapies have been proposed, offering new opportunities in tackling these conditions. Pre-clinical animal models define an essential component of the research process and are implemented to not only investigate the safety and efficacy of proposed therapies but also allude to the mechanisms of action. The need to rapidly and reproducibly assess optimal delivery routes, cell doses, tissue engraftment and cellular bio-distribution patterns, whilst also complying with the NC3Rs to minimise the number of animals inflicted, has driven the need for minimally invasive techniques to monitor in vivo cell fate. The combined use of magnetic resonance imaging (MRI) and superparamagnetic iron oxide nanoparticles (SPIONs) has been proposed as one such minimally invasive strategy [10], the feasibility of which is explored in a osteochondral pre-clinical sheep model and reported in this manuscript.

SPIONs are well known for their clinical application as a T_2_-weighted MRI contrast agent and have been used in the diagnosis of a wide range of diseases and injuries [21]. Early successful adopters of MRI cell tracking protocols utilised either Endorem or Resovist both of which are FDA-approved MRI contrast agents to label stem cells for follow on MRI tracking in a number of organs including the articular knee joint [22–25]. As of 2009, the manufactures of these compounds withdrew both products from the market siting economic reasons [24, 26, 27]. As a result, a number of off-label (e.g. Feraheme), in-house or commercial SPIONs have been investigated showing promise in tracking cells in a wide range of clinical indications such as neural regeneration, pancreatic islet transplantation and renal regeneration [28].

For the first time, we introduce the commercially available SPION, Nanomag-D, as a potential cell tracking contrast agent. This particle has been implemented in the development of a pioneering technique whereby the particle, Nanomag, is used to remotely activate key mechanotransduction pathways involved in osteogenic differentiation of MSCs using an external magnetic field [15]. This technique, known as magnetic ion channel activation (MICA), has recently been employed in a pre-clinical sheep model of bone injury supporting the development of an injectable therapy for non-union bone fractures [19]. In future work, we plan to utilise MICA in the development of an injectable cartilage repair therapy by introducing mechanical influences otherwise lacking in standard cartilage therapies. The results presented in this manuscript are significant in that the optimal contrast-forming dose of Nanomag (25 μg/ml) is aligned with in vivo MICA activation doses utilised in the pre-clinical study [19]. Therefore, Nanomag can potentially be taken forward as a dual MRI and activation agent and applied to further our understanding of repair mechanisms by mapping the location of Nanomag-labelled cells relative to repair sights by MRI with little manipulation of the labelling protocol.

Studies have reported poor cellular uptake of dextran-coated SPIONs by non-phagocytic cells such as MSCs thereby limiting their application as MRI cell tracking agents [29, 30]. In line with our result, we observe limited uptake of Nanomag, a 250-nm dextran-coated particle, resulting in poor MRI contrast in vitro regardless of incubation time and labelling concentration. Transfection agents such as poly-l-lysine (PLL), protamine sulfate and lipofectamine have played a crucial role in enhancing the uptake of SPIONs (such as the FDA-approved particles mentioned earlier) to detectable levels for MRI tracking applications [29, 31–33]. These cationic compounds function by forming positively charged complexes with SPIONs to encourage electrostatic attraction with the negatively charged cell membrane [31]. Although significant improvements in uptake efficiency have been reported, these compounds are associated with dose-dependant toxic effects [30]. In this study, a novel cell-penetrating peptide P21-8R intended to enhance the uptake of Nanomag by oMSCs using a technique known as GET (glycosaminoglycan-binding enhanced transduction) has been investigated [20]. The system, developed by our group, functions to improve the activity of standard cell-penetrating peptides to ultimately enhance intracellular delivery of cargos. It involves the interaction of the peptide P21-8R with cell membrane heparan sulfates to promote endocytosis [20]. We further demonstrate a significantly positive shift in the charge of Nanomag once complexed further promoting cell interactions towards improved uptake. Importantly, this approach is considered safe and does not affect cell proliferation and viability [20].

Little or no adverse effects have been reported with SPION labelling in terms of cell proliferation, viability and differentiation potential. SPION toxicity is often correlated to dose, composition and the immediate microenvironment of the particle all of which can trigger unwanted toxic effects either directly to implanted cells or the surrounding tissue [34]. Here we demonstrate no diminished cell viability, proliferation and differentiation potential across six sheep donors when labelled with GET-Nanomag in vitro. Conflicting reports have however raised concern regarding the chondrogenic differentiation potential of SPION-labelled MSCs with studies reporting either impaired chondrogenesis [35–37] or no effect at all [25, 28]. This is thought to be a dose-dependant effect accounting for the contrasting reports although further analysis is required [38, 39]. In our study, we observed no compromised chondrogenesis of autologous oMSCs when labelled with GET-Nanomag in vitro. Furthermore, CRP (c-reactive protein) levels in vivo were determined to be within normal range in both the acute and chronic injury model implying that the delivery of GET-Nanomag-labelled cells has not elicited an unwanted short-term immune response. This data provides further support for the in vivo use of this magnetic particle system in the development of an osteochondral therapy.

We report the detection of GET-Nanomag-labelled MSCs in the articular knee joint of sheep 7 days post-delivery using a 0.25-T veterinary MRI scanner with results validated on a 3-T clinical grade scanner post sacrifice. Labelled cells are identified as hypointense regions, made particularly obvious where there are “blooming” artefacts, a phenomenon whereby the signal from the SPION extends far beyond the size of the particles due to the high susceptibility of large concentrations of ferrous material. This allows for high concentrations of SPION-labelled cells to be easily and practically identified against anatomical tissue [10]. MRI results on day 7 revealed no hypointense regions at the OCL injury site in either the acute or the chronic injury models implying that cells have not homed to the site of injury. Furthermore, this technique allows for differences in the distribution patterns of labelled cells between the two models to be observed. Characteristic hypointense blooming is seen in the injured leg of the chronic model but not in the acute model despite detection of cells in the control leg of the acute model. Although the blooming phenomenon facilitates easy detection of implanted cells, it may result in key anatomical structures being lost as can be seen on the 0.25-T images. This makes it extremely difficult to determine the exact location of SPION-labelled cells and is considered a limitation of the study. To facilitate clinically relevant scan durations, different protocols are used on the two MRI scanners resulting in visually different contrast, and marginally less influence of the blooming artefact at 3 T despite the higher field. Despite the different weighting of the images, the effect of the SPIONs is similar since the signal loss caused by dephasing will dominate most gradient and spin echo sequences.

We hypothesise that the variations in the distribution patterns observed in the chronic and acute injury models are related to the inflammatory environment of the injured joint. In this case, the creation of the OCL defect triggers an inflammatory response which is associated with the release of inflammatory mediators, enhanced cellular infiltration and increased monocyte and macrophage content. This is greatest in the acute post-injury period (up to 1 week) but will be sustained at lower levels thereafter [40]. It is therefore suggested that the heightened inflammatory environment of the acute injury results in increased macrophage recruitment which acts to clear implanted cells prior to sacrifice. This is corroborated by immunohistochemical analysis where a distinct lack of CD45-positive leukocytes is observed in the acute model [41]. In the chronic injury model however, where cells are minimally invasively delivered to the knee joint 4.5 weeks post injury when inflammatory levels are reduced, cells remain within the synovial joint as detected by MRI and by histology. This implies that cells are not cleared from the knee joint and are instead captured and localised by a complexed community of macrophages found in the synovium. CD45 is a transmembrane glycoprotein representative of leukocytes which are typically characterised as white blood cells or immune cells including macrophages and monocytes and are recruited as part of the inflammatory response to injury. In studies investigating the effects of nanoparticles on the inflammatory process of the articular knee, it has been shown that nanoparticles are engulfed by monocytes or macrophages, particularly by those residing in the synovial lining and have been detected up to 14 days post intra-articular implantation [42] in a number of small [43] and large animal studies [44]. Similar observations are made in this study where Nanomag-labelled cells are observed along the synovial lining.

The migration and adhesion of MSCs to a cartilage injury is dependent on multiple factors including the secretion of chemotactic factors by damaged cartilage or synovial tissues, the expression of chemotactic receptors by MSCs, the adherence properties of the tissue/cartilage and the mechanical shear stresses in the surrounding environment [45]. Whilst studies demonstrating cell homing to the injury site following in vivo intra-articular delivery have been reported, it has been noted that the ratio of cells homed to the desired site is limited. This is a cause of clinical concern as it can impact therapeutic outcome and is therefore an area of continuous research [46, 47]. It has been shown that the exposure of MSCs to an inflammatory and/ a hypoxic environment can influence the expression of migratory factors of MSCs [45, 48]. Furthermore, many studies have observed a preferential accumulation of delivered MSCs to the synovium which may be due to the release of an alternative chemotactic release profile [45]. Another explanation for the observed improved adhesion of MSCs to the synovium could be that the mechanical forces experienced in the joint mobilise the MSCs to the synovium and that cells are more likely to attach to a rough surface like the synovium than to a smooth surface like cartilage.

The majority of cartilage and osteochondral tracking studies reported in the literature have focused on small animal models of cartilage injuries with the mode of delivery mimicking MACI or MASI (matrix-assisted chondrocyte or stem cell implantation). In these systems, cells are localised to the injury site, fixed in place and the degree of repair monitored using a 7- or 11-T MRI scanner. These studies have been successful in generating evidence of scaffold failure and scaffold engraftment by understanding MRI signal characteristics relating to particular events and have also demonstrated evidence of engraftment of stem cells to the defect site by MRI [14]. In our opinion, it is not practical to design pre-clinical tracking approaches in small animal models to high strength scanners knowing that such resolution will not be achieved in pre-clinical veterinary scenario when translating to clinically relevant large animals such as sheep without substantial cost and capital investment. This is not to say that high-strength scanners should not be used for small animal models as they do undoubtedly offer a powerful means of gathering data linked to mechanism of action, etc., in the early stages of therapy development. It is also important to note that conclusions from this study were drawn immediately from the 0.25-T veterinary MRI scanner and only validated some time later on the 3-T clinical scanner and by histology. This supports the application of this system as a practical means of generating data in large animal pre-clinical studies without the need for high-strength scanners. Furthermore, the larger extent of blooming seen on the 0.25-T images makes detection, if not localisation, of the SPIONs easier without the capital costs associated with the high-field MRI scanners.

## Conclusion

To conclude, this study demonstrates the feasibility of tracking autologous MSCs in a large animal osteochondral injury model using both low-field veterinary and high-field clinical MRI scanners. We prove the use of Nanomag in conjunction with the cell-penetrating peptide system as a plausible tracking agent in cell-based therapies. Finally, this study further demonstrates that MSC cell behaviour and potency vary with treatment regimens in clinical scenarios.

## Abbreviations

AA: Antibiotic and anti-mycotic
CRP: c-reactive protein
FDA: Food and Drug Administration
GET: Glycosaminoglycan-binding enhanced transduction
H&E: Haematoxylin and eosin
HCL: Hydrochloric acid
LG: l-glutamine
MACI: Matrix-assisted chondrocyte
MASI: Matrix-assisted stem cell implantation
MFC: Medial femoral condyle
MICA: Magnetic ion channel activation
MRI: Magnetic resonance imaging
MSC: Mesenchymal stromal cell
MSME: Multi-slice multi-spin echo
NC3R: National Centre for the Replacement, Refinement and Reduction of Animals in Research
OA: Osteoarthritis
OCL: Osteochondral lesion
PLL: Poly-l-lysine
RBC: Red blood cells
SFM: Serum-free media
SPION: Superparamagnetic iron oxide nanoparticle
